# A citizen science supported study on seasonal diversity and monoflorality of pollen collected by honey bees in Austria

**DOI:** 10.1038/s41598-019-53016-5

**Published:** 2019-11-12

**Authors:** Robert Brodschneider, Kristina Gratzer, Elfriede Kalcher-Sommersguter, Helmut Heigl, Waltraud Auer, Rudolf Moosbeckhofer, Karl Crailsheim

**Affiliations:** 1 University of Graz, Institute of Biology, Universitätsplatz 2, 8010 Graz, Austria; 2 Austrian Agency for Health and Food Safety, Ltd., Institute for Seed and Propagating Material, Plant Protection Service and Apiculture, Department for Apiculture and Bee Protection, Vienna, Austria

**Keywords:** Biodiversity, Entomology

## Abstract

Austrian beekeepers participated in the “C.S.I. Pollen” study as citizen scientists and collected pollen from honey bee colonies in hive mounted traps every three weeks from April to September in 2014 and 2015 to uncover the seasonal availability of pollen sources for bees. 1622 pollen samples were collected and analysed using palynological light microscopy to the lowest taxonomic level possible. For 2014 and 2015 combined, 239 pollen types from more than 85 families were detected. ‘Various unknown’ species, *Taraxacum*-form and *Plantago* spp. were the pollen types collected by the majority of colonies (occurrence), whereas the most pollen grains collected were from *Trifolium repens*-form, *Plantago* spp. and *Salix* spp. (abundance). In spring, trees were found to be the most abundant pollen source, whereas in summer herbs dominated. On average, a colony collected pollen from 16.8 ± 4.7 (2014) and 15.0 ± 4.4 (2015) pollen types per sampling. Those numbers, however, vary between sampling dates and indicate a seasonal pattern. This is also supported by Simpson’s diversity index, which was on median 0.668. In both years, 50.0% of analysed pollen samples were partially (>50%) and 4.2% were highly monofloral (i.e. containing >90% of one pollen type). Prevalence of monofloral pollen samples peaked at the beginning and the end of the season, when pollen diversity was the lowest.

## Introduction

The honey bee is a polylectic organism with pollen being the only important natural source of proteins and lipids. It is essential for brood rearing, adult bee’s development and affects honey bee health^1–5^. Pollen differ in their nutritional value to bees and a highly diverse or mixed diet is therefore regarded as more beneficial for honey bees than monofloral pollen diets^6–11^. As a consequence, the protection of floral diverse environments is an important factor to enhance the fitness and population stability of social bees^11^.

Information on the forage sources visited by a honey bee colony can be obtained by pollen analysis (palynology) of the pollen found in honey (melissopalynology)^12,13^, by analysis of corbiculate pollen loads of returning foragers or pollen stored as beebread. As the pollen in honey can also originate from nectar sources, only analysis of pollen loads collected by pollen foragers or beebread describes the available sources of protein to a honey bee colony that can be converted to body tissue or brood food^14,15^.

The pollen collected by honey bees has been extensively studied in other countries before. The majority of these investigations determined botanical origin with palynological light microscopy, though also molecular next generation sequencing or multi-locus metabarcoding methods exist^16–18^. According to the addressed research question, previous investigations of the bees’ pollen spectrum were often temporally and spatially limited to certain habitats like agricultural sites^15,19–25^ or urban and suburban areas^17,26^. Other studies compared the pollen availability of different landscapes^27–29^. In this first Austrian study on honey bee pollen foraging ecology we aim for a wide range of different sample locations all over the country and to cover a long period of the honey bee foraging season. We approach this by the involvement of beekeepers who are voluntarily supporting research as citizen scientists. Sample sites are citizen scientists’ private apiaries, which results in heterogenous landscapes in the flight range of the honey bee colonies. Furthermore, this entails that the sampling locations are not actively selected for special or extreme habitats.

Citizen science is understood as a collaborating network of people, who contribute to science with their dedication. They not only provide experimental data and facilities for researchers, but their output also raises new questions^30,31^. In our study, volunteer beekeepers helped in collecting pollen samples to learn about the pollen diversity of their honey bee colonies. The international C.S.I. Pollen study (citizen scientist investigation on pollen diversity forage available to honey bees), coordinated by the honey bee research association COLOSS, of which this study is the Austrian branch, was comprised of two levels: in many European countries citizen scientists were asked to collect pollen every three weeks and to identify the number of different colours in the pollen sample to roughly estimate pollen diversity^32,33^. That study involved the recruitment and training of citizen scientists in regard of installing pollen traps, sampling, processing the pollen samples and cleaning the equipment to prevent sample contamination. In some countries citizen scientists were also equipped with sample bags and instructions on how to prepare and conserve samples for later palynological analysis in a laboratory. Therefore, comparable results to this Austrian study are available from seven locations in Liguria, Italy^32,34^.

The cooperation with citizen scientists enabled us to collect pollen with pollen traps at sampling locations all over Austria to study pollen availability for honey bee colonies. This for the first time provides the possibility to identify important pollen sources for honey bee nutrition in Austria, to discuss the diversity of pollen available to honey bee colonies and to detect potential seasonal shortages in the number of different pollen sources.

## Material and Methods

### Sampling locations, sampling series and pollen trapping

Within the COLOSS C.S.I. Pollen project, Austrian beekeepers were recruited as citizen scientists (CSs) by giving talks, writing articles in the Austrian beekeeping journal and propagation via internet. The study was carried out in accordance with all relevant guidelines and regulations. Participants were provided with a step by step picture manual (pamphlet) and instructed to collect pollen from pollen traps on up to three non-migratory bee colonies (numbered one, two and three) of the same environment (apiary) at one sampling date^33^. The colonies and apiary locations remained unchanged all season. Each sampling was scheduled within a four-day time frame (Thursday - Sunday) from which the CSs chose a day suitable for them to collect the pollen samples (Table 1). The communication process between the CSs and the study coordinators was organized using the open source survey tool LimeSurvey^TM^ version 1.91 + (LimeSurvey GmbH). By establishing a communication routine covering the time prior to, during and after each sampling process, the CSs provided information on sampling parameters (actual date of sampling, duration of sampling and GPS location). Pollen traps were closed for preferably one day or up to three days to provide the minimum sample size of pollen required (20 g).

**Table 1:** Sampling dates, number of locations and number of analysed pollen samples in 2014 and 2015.

Sampling date identifier	2014			2015			Both years
	Date	Locations	Pollen Samples	Date	Locations	Pollen Samples	∑ Pollen samples
April I	3–6 April	24	70	2–5 April	14	40	110
April II	24–27 April	32	93	23–26 April	34	97	190
May	15–18 May	36	102	14–17 May	36	104	206
June I	5–8 June	38	107	4–7 June	35	100	207
June II	26–29 June	31	91	25–28 June	36	105	196
July	17–20 July	33	97	16–19 July	31	92	189
August I	7–10 August	31	87	6–9 August	30	86	173
August II	28–31 August	28	79	27–30 August	35	104	183
September	18–21 September	28	78	17–20 September	32	90	168
∑ Pollen samples			**804**			**818**	**1622**

**Figure 1:**
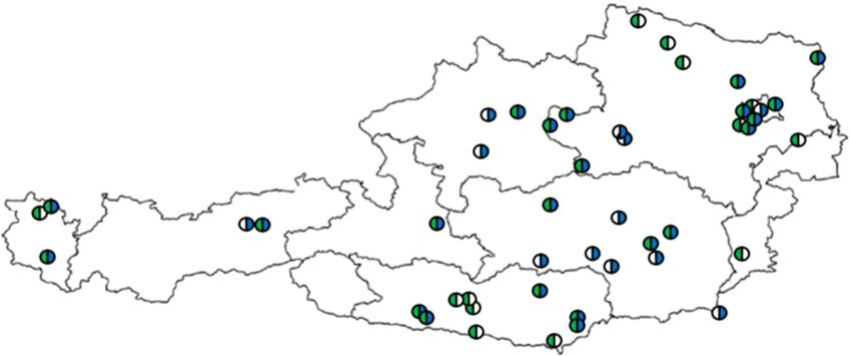
Sampling locations (citizen scientists’ apiaries) in 2014 (green) and 2015 (blue).

The management of bee colonies, the installation of pollen traps and the collection, processing and storage of samples were carried out independently by the CSs. Sampling receptacles with pre-printed labels were provided to the participants. The overall participation in this study was higher than the capacity for sample analysis and therefore only the most complete sample series from all locations were analysed. During the two sampling seasons (i.e. in 2014 and 2015) 18 complete sample series (nine samplings × three colonies = 27 samples) were analysed. Further sample series were chosen for analysis based on high number of samples, but also on regional coverage. Hence, in both years 1622 pollen samples from all Austrian federal states were successfully examined (Table 1). Those were collected by 49 different CSs at 51 sampling locations (38 and 36 locations for 2014 and 2015, respectively), whereby 23 of these sites were sampled in both years (Fig. 1). Per location and year the average number of samples examined by light microscopy was 21.9. The minimum analysed number of samples from one sampling location in one year was seven (in 2014).

### Palynological analysis and assignment to pollen types

**Figure 2:**
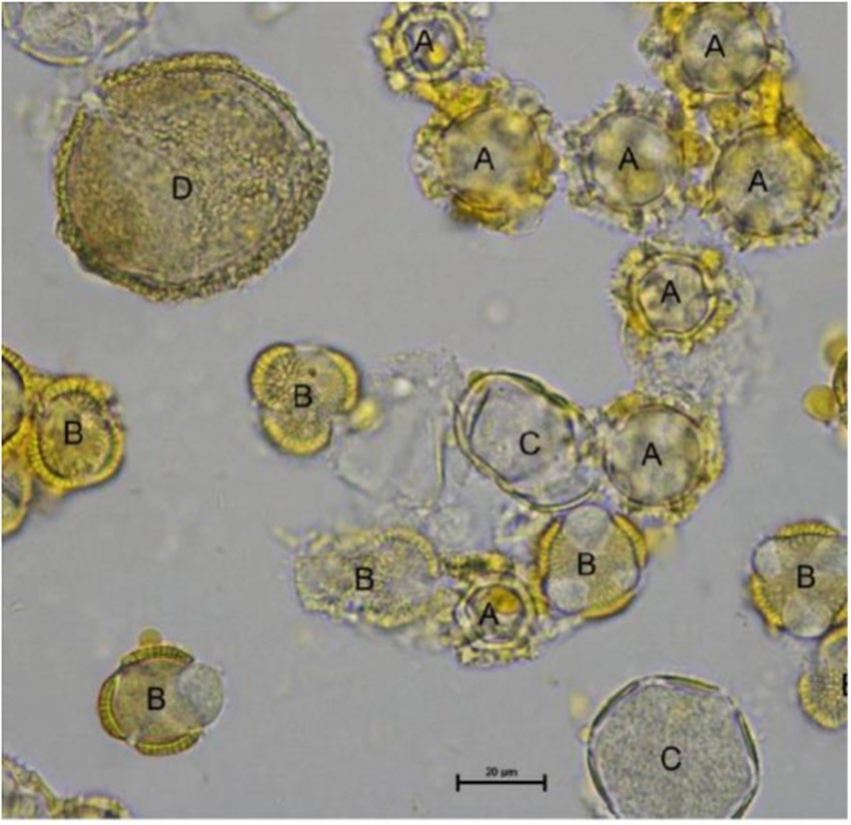
Example for the analysis of pollen types by light microscopy. The sample originates from Austria (Styria) and was collected in late April. Pollen from (**A**) *Taraxacum*-form, (**B**) Brassicaceae, (**C**) Lamiaceae-form and (**D**) unknown species are shown.

Approximately 20 g of the pollen collected per colony were first dried and then stored in the freezer in labelled sampling receptacles by the CSs. Defrosted samples were sent to our laboratory for palynological analysis at the end of the season. Microscopic palynological analysis of pollen pellets was conducted following the method of Barth *et al*.^35^ as also applied in Brodschneider *et al*.^36^. Briefly, depending on the weight and the dryness of the pollen samples, distilled water was added and the suspension was stirred, applied on a microscope slide using a micropipette and dried on a heating plate. In a final step, the sampling slides were covered with glycerine jelly and further dried for 24 h prior to analysing. According to the studies by Dimou *et al*.^37^ and Lau *et al*.^38^ 5–10% of total trapped pollen and the palynological analyses of 500 pollen grains are sufficient to provide significant analytical results as well as information on the main pollen sources of the area. Therefore, 500 pollen grains were analysed using a light microscope at a magnification of 400x–1000x under oil immersion. Pollen grains were identified by comparing them to circa 2000 full records reference specimens from the Austrian pollen database at ponet.ages.at. This database allows assignment of pollen grains to different taxonomic levels according to a six-digit pollen code and light microscopy photographs (Fig. 2).

Pollen types with similar morphology, which were not further distinguishable with light microscopy, were allocated to the next higher taxonomical category (genus, family) or form a cryptic group of several closer related genera (e.g. *Malus* spp., *Pyrus* spp., *Crataegus* spp. as members of the Rosaceae-plant family). While all rarely appearing pollen forms not yet listed in the pollen database were summarized as ‘unknown’, so far unidentified pollen forms with a higher frequency were measured microscopically and classified with a six-digit pollen-code (e.g. ‘unknown 776131’). All pollen grains not allocatable, due to a failed reaction during the preparation process, were defined as ‘not swollen’.

### Data analysis

The total number of different pollen types and the mean (±standard deviation) number of different pollen types were both noted for each sampling date. Pollen forms were, if possible, assigned to plant families and growth-form (herbs, trees, shrubs, climbers/creepers, unknown growth-form, see Table S1 for a detailed list). Samples containing a relative amount of more than 50% of the same pollen type were defined as partially monofloral pollen samples, whereas samples consisting of more than 90% of one botanical source were termed highly monofloral pollen samples. For both types of monofloral pollen samples, i.e. partially and highly monofloral, relative frequencies per sample date were calculated. To test for significant differences in the proportion of partially and highly monofloral pollen samples found across the nine sampling dates a Pearson’s Chi-Square test was conducted.

The term ‘occurrence’ expresses the number of samples, tested positive for a specific pollen type; the value never exceeds the total number of samples (n = 1622). To calculate the ‘abundance’ of pollen types, calculations were based on the number of analysed pollen grains. Different pollen types were, similar to Dimou and Thrasyvoulou^15^, classified into frequency categories based on overall relative abundance per sample date (<1%, 1–5%, >5–20% and >20%) to distinguish between rare or commonly appearing pollen taxons. Simpson’s diversity index (D) was used to assess the pollen diversity for each sample and was calculated based on the formula $D=1-[\frac{\sum n(n-1)}{N(N-1)}]$. Where *N* represents the total grain number of a sample, while *n* describes the number of pollen grains, assigned to a certain pollen form.

## Results

In 2014, 804 pollen samples from 38 locations were analysed, while in 2015 818 pollen samples were collected from 36 locations. In 90.6% of the sampling events, a sufficient amount of pollen for palynological analysis could be collected from all three study colonies at the respective apiary, whereas in 6.4% of cases only pollen samples from two colonies, and in 3.0% only samples from one colony was available for analysis. Overall, it was possible to differentiate between at least 203 pollen types in 2014, 207 in 2015, and at least 239 pollen types from over 85 taxonomic plant families for the whole observational period (i.e. 2014 and 2015). Over all sampling dates and locations, the mean number of plant families was 50.4 (±8.5) and 48.9 (±7.1) for 2014 and 2015, respectively. Asteraceae, Fabaceae and Rosaceae were identified as the most occurring taxonomic families. Focusing on a single sampling site and a complete one year sampling period, the highest number of different botanical pollen sources was 105, whereas on average it was 84.9 (±9.8) per site. The mean number of pollen types, identified in one sample was 16.2 (±5.7). In the first year of data collection, we were able to allocate 185 (=91.1%) and in 2015 190 (=91.8%) pollen types to their systematic botanical sources (species, genus, family level or cryptic group of pollen types). Depending on their characteristics as mentioned in Material and Methods, the remaining pollen forms were either summarized as ‘various unknown’ species, ‘unknown’ species with a six digit code, or defined as ‘not swollen’.

**Figure 3:**
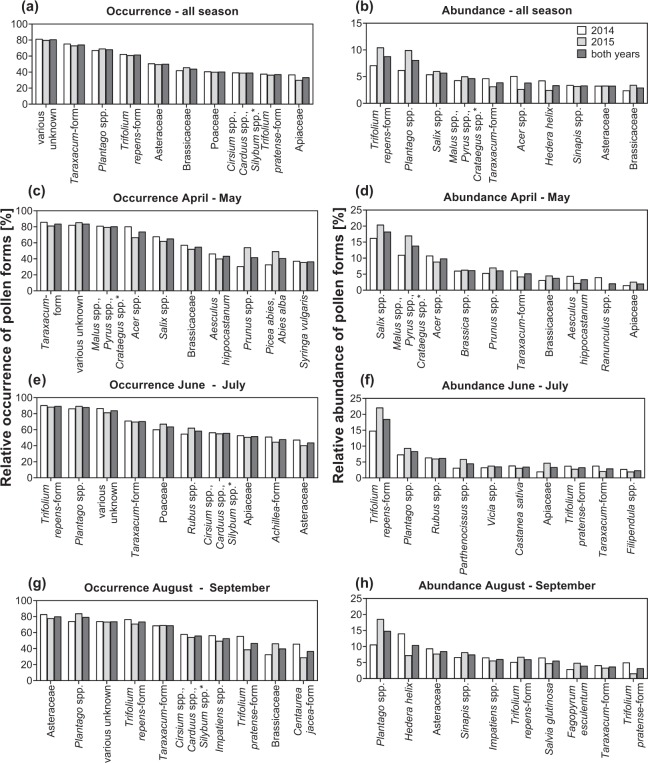
Occurrence and abundance of the ten most important pollen forms ranked by the results for both years (n = 1622). (**a**,**c**,**e,g**) Show the relative amount of positively tested pollen forms per number of samples (occurrence). (**b,d,f,h**) Show abundance (based on number of pollen grains) of pollen forms. (**a,b**) Results for all nine sampling dates, (**c,d**) for spring, (**e,f**) for (early) summer and (**g,h**) for late summer and autumn, are represented. Sample sizes for each sampling date are shown in Table 1. *Form cryptic groups, not further differentiable by light microscopy. The complete list of every pollen type’s occurrence and abundance is listed as supplement (Tables S1, S2).

The occurrence and abundance of pollen forms were calculated, based on the number of sample sizes and pollen grains, respectively. A complete list of every pollen type’s occurrence and abundance is listed as supplement (Tables S1 and S2), while in Fig. 3 the top ten pollen taxons for all seasons and further clustered by sampling dates (April – May, June – July, August – September) are shown. Summarized for both full seasons, ‘various unknown’ (80.7%) species, *Taraxacum*-form (74.2%), *Plantago* spp. (68.3%), *Trifolium repens*-form (61.7%) and pollen types of the family Asteraceae (50.2%) had the highest occurrence. Regarding abundance, *Trifolium repens*-form (8.8%), *Plantago* spp. (8.1%), *Salix* spp. (5.7%), the cryptic group of *Malus* spp./*Pyrus* spp./*Crataegus* spp. (4.7%) and *Taraxacum*-form (3.9%) dominated (Fig. 3a,b). Overall, the ten most abundant pollen types collected by honey bees in Austria accounted for up to 48.0% of the total number of analysed pollen grains.

In spring (April – May), 168 different pollen forms were collected and *Taraxacum* (83.8%), ‘various unknown’ (83.8%) species, pome fruit trees (80.4%), Salix spp. (73.9%) and Acer spp. (65.2%) had the highest occurrence, while Salix spp. (18.2%), pome fruit trees (13.9%), Acer spp. (9.9%), Brassica spp. (6.2%) and Prunus spp. (6.2%) were the most abundant pollen types (Fig. 3c,d). During (early) summer (June – July) Austrian honey bees foraged on 189 different pollen sources. Based on their occurrence, the most dominant pollen types were *Trifolium repens*-form (89.5%), *Plantago* spp. (88.0%), ‘various unknown’ (84.1%) species, *Taraxacum* (70.6%) and pollen types from the family Poaceae (63.9%). *Trifolium repens*-form (18.5%), followed by *Plantago* spp. (8.4%), *Rubus* spp. (6.2%), *Parthenocissus* spp. (4.5%) and *Vicia* spp. were the most abundant pollen types (Fig. 3e,f). During (late) summer (August – September), 145 pollen sources were identified and pollen types from the family Asteraceae (80.2%), *Plantago* spp. (79.4%), ‘various unknown’ (73.9%) species, *Trifolium repens*-form (73.7%) and the cryptic group *Cirsium* spp./*Carduus* spp./*Silybum* spp. (56.1%) showed the highest occurrence. Most abundant pollen types were *Plantago* spp. (14.9%), *Hedera helix* (10.4%), pollen from the family Asteraceae (8.5%), *Sinapis* spp. (7.5%) and *Impatiens* spp. (6.1%) (Fig. 3g,h).

**Figure 4:**
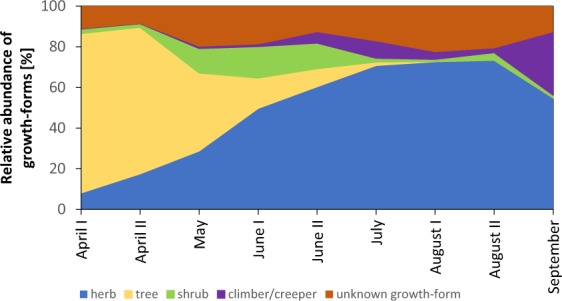
Pollen grain abundance according to growth-form of plants. Percentage of pollen types collected by honey bees in Austria during two consecutive years (2014 and 2015) is shown (n = 810604 pollen grains from 1622 samples).

From the 239 pollen species, 49.8% of pollen grains came from herbs, followed by trees (15.5%), shrubs (12.6%) and climbers/creepers (3.3%; Fig. 4). The remaining pollen grains were summarized as ‘unknown growth-form’ and correspond to 18.8% of pollen types. Trees were most abundant in spring, providing 59.7% of analysed pollen grains in April – May. In the following months (June – August) herbs showed the highest abundance, while the abundance of pollen grains from trees sharply declined. In September pollen grains from trees almost completely vanished and herbs (54.9%) and climbers/creepers (31.0%) provided the most pollen.

**Figure 5:**
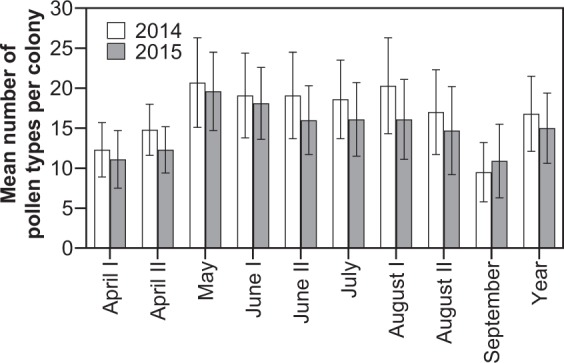
Mean (±SD) number of pollen types collected per honey bee colony at nine sampling dates (between early April and late September) during the two observational periods 2014 and 2015. The last two bars represent the results for the whole season (for sample sizes see Table 1).

On average, we found between 9.5 (±3.7; September 2014) and 20.7 (±5.6; May 2014) different pollen types per colony at a sampling date (Fig. 5). While at the beginning (early April) and the end of the season (September) the mean numbers of pollen types per colony were similarly low, the highest ones were observed in May followed by June. The mean number of different pollen types almost doubled within the first three sampling dates in both years and a mean of constantly more than 15 different pollen sources was observed during the summer months. Summarized for the whole observational period, the average number of pollen types collected per colony was 16.8 (±4.7) in 2014 and 15.0 (±4.4) in 2015, respectively (Fig. 5).

**Figure 6:**
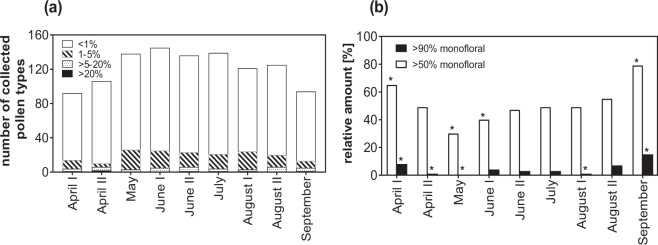
(**a**) Number of pollen types honey bees collected in Austria during the apicultural periods of 2014 and 2015 combined including their frequency categories <1%, 1–5%, >5–20% and >20% based on overall relative abundance per sample date; (**b**) partially (>50%) and highly (>90%) monofloral pollen samples and their relative frequency to the total number of samples per sampling date are shown (for sample sizes see Table 1). * Indicates a significant difference to the expected value (i.e. a standardized residual ≥ ±2).

Over the two-year period, it was possible to discriminate between at least 239 different botanical sources, with a minimum of 92 recorded in early April and the maximum number of 145 found in early June (Fig. 6a). Pollen sources with abundances higher than 20% per sampling date were relatively rare and made up the smallest group, while most pollen forms were found with abundances lower than 1%.

### Diversity during the apicultural season

**Figure 7:**
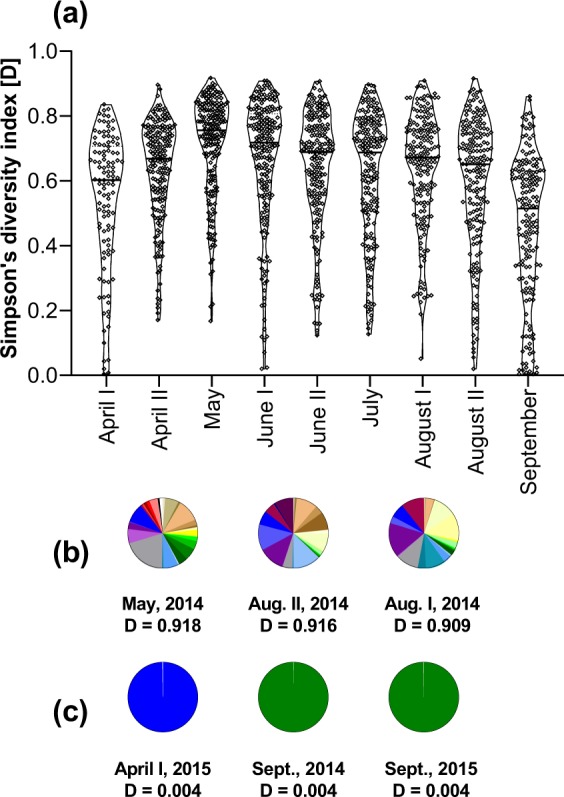
(**a**) The violin plots describe the variation of Simpson’s diversity index D during both apicultural periods 2014 and 2015 combined. Horizontal lines mark the medians and the diamond symbols represent the probability density of analysed samples (for sample sizes see Table 1); (**b**) Pie charts examplarily showing pollen type composition of the three samples with the highest diversity indices, found in May 2014 (33 pollen types), August I 2014 (27 pollen types) and August II 2014 (29 pollen types). Different colours indicate different pollen types. (**c**) The three samples with the lowest diversity indices, found in April I 2015, September 2014 and September 2015 which consisted of only 2 species each: *Salix* spp. (blue) and unknown; *Hedera helix* (green) and Asteraceae; *Hedera helix* (green) and unknown, respectively.

In Fig. 7a the seasonal Simpson’s diversity (D) indices of analysed pollen samples over both investigated apicultural seasons are represented. Data revealed a median of 0.668 over both seasons. Focusing on sample level, the lowest D of 0.004 were observed in April 2014 and September 2014/2015 and the highest D of 0.918 was identified in May 2014 (Fig. 7a,c). The highest median pollen diversity among different sampling dates was found in May with 0.756 (Fig. 7b). Further, results indicate a seasonal pattern with diversities peaking during late spring and the summer months. The distribution of different pollen sources in samples with the highest and lowest diversity indices are exemplarily shown in Fig. 7(b,c). Whereas the highly diverse pollen samples were comprised of 27 to 33 pollen types (Fig. 7b), the samples with the lowest D consisted of mainly one pollen type (99.8%) plus one single pollen grain from another botanical source (Fig. 7c).

### Nearly monofloral pollen samples

Samples containing a relative amount of more than 50% of the same pollen type were defined as partially monofloral pollen samples. This was the case for 46.3% of the samples collected in 2014 (n = 804). Twenty-five (3.1%) pollen samples in 2014 consisted of more than 90% of one botanical source, hereafter termed as highly monofloral pollen samples. Similarly, in 2015 439 (53.7%) of 818 pollen samples contained more than 50% of the same pollen type and 43 (5.3%) more than 90% of one pollen type. Summarized for both years, 50.0% of analysed pollen samples (811 out of 1622) were partially and 4.2% were highly monofloral. The ten most abundant pollen types identified, making up partially monofloral (67 different pollen types) and highly monofloral pollen samples (13 pollen types) are shown in Table 2, a complete list is shown in Table S3. For both years combined, *Trifolium repens*-form, followed by *Salix* spp., *Plantago* spp. and *Hedera helix* were found to be the most common partially monofloral (>50%) pollen sources. *H. helix* was also found to be the most prominent pollen taxon for highly monofloral samples (>90%) (Table 2). During the observational period, between 29.6% (May) and 79.2% (September) of samples were identified as partially monofloral (Fig. 6b). Highly monofloral pollen samples were most frequently found in September, when they made up 15.5% of 168 pollen samples (Fig. 6b). The relative amount of partially (i.e. more than 50%) monofloral pollen is not evenly distributed across the nine sampling dates (Pearson’s Chi-Square: χ^2^ = 112.6, df = 8, p < 0.001). While the number of partially monofloral pollen samples is significantly higher than expected in early April (standardized residual *r* = 2.2) and September (*r* = 5.3), it is significantly lower than expected in May (*r* = −4.1) and June I (*r* = −2.1, Fig. 6b). Similarly, the relative amount of highly (i.e. more than 90%) monofloral pollen is not evenly distributed across the sampling season as well (χ^2^ = 81.7, df = 8, p < 0.001). In early April (*r* = 2.0) and September (*r* = 7.1) the number of highly monofloral pollen samples is significantly higher than expected, whereas it is significantly lower than expected in late April (*r* = −2.5), May (*r* = −2.6) and early August (*r* = −2.3, Fig. 6b).

**Table 2:** Number and frequency of partially (>50%) and highly (>90%) monofloral pollen samples collected in Austria in 2014 (n = 804) and 2015 (n = 818 pollen samples). *Form cryptic groups, not further differentiable by light microscopy. All pollen forms found to be monofloral are listed as supplement (Table S3).

>50%			>90%		
Pollen type(s)	Number of samples	Frequency [%]	Pollen type(s)	Number of samples	Frequency [%]
*Trifolium repens*-form	86	5.30	*Hedera helix*	18	1.11
*Salix* spp.	85	5.24	*Salix* spp.	10	0.62
*Plantago* spp.	72	4.44	*Trifolium repens*-form	7	0.43
*Hedera helix*	53	3.27	*Impatiens* spp.	6	0.37
*Malus* spp., *Pyrus* spp., *Crataegus* spp.*	46	2.84	*Plantago* spp.	6	0.37
*Sinapis* spp.	41	2.53	*Sinapis* spp.	6	0.37
*Acer* spp.	35	2.16	*Vicia* spp.	5	0.31
*Salvia glutinosa*	29	1.79	*Castanea sativa*	3	0.18
Asteraceae	23	1.42	*Rubus* spp.	2	0.12
*Brassica* spp.	23	1.42	*Trifolium pratense*-form	2	0.12

## Discussion

The consumption of a diverse pollen diet is crucial for a healthy nutrition of honey bees^2,7,8,39^. So far, no comprehensive investigations neither on pollen diversity nor on corresponding seasonal patterns of pollen sources for honey bee pollen foraging ecology existed for Austria. To fill this gap and to facilitate the understanding of bee health and colony development, pollen samples collected from citizen scientists from all over Austria were analysed by experts using palynological light microscopy. The involvement of citizen scientists enabled us to include many samples from different locations in our investigation. This meant that the apiary locations were not selected for special landscape traits as in previous studies, but comprised a real situation at often year-long established apiaries. We obtained samples from all nine Austrian federal provinces, with a majority coming from Eastern Austria, which also reflects the distribution of beekeeping intensity in Austria^40^. As retention is important in citizen science investigations^41^, we tried to keep motivation high, by providing detailed palynological results of their sampling location to the citizen scientists. This underlines the great interest of participating beekeepers in learning about phenology of flowering plants in the vicinity of their colonies.

One aim of this investigation was to learn about the pollen collected by bees at typical Austrian apiary locations, especially from other plants next to those commonly listed as important pollen sources for bees^42,43^. According to Fischer *et al*.^44^, there are around 3300 higher plant species in Austria (including South Tyrol and Liechtenstein) from 152 taxonomic families plus circa 600 ornamental, agricultural or extinct plants. In 1622 pollen samples we identified 239 different pollen types from at least 85 taxonomic families, which corresponds to 6.1% of the higher plant species summarized by Fischer *et al*.^44^. As shown by Ollerton *et al*.^45^ about 78% of plant species in temperate climates are pollinated by animals, though the honey bee is only one of the many pollinating species. The number of different pollen sources for bees detected in our study therefore is very likely an underestimation, due to numerous reasons: our first sampling dates were set in April, where some important early pollen sources already ceased blooming; the sampling intervals were three weeks or even longer in case citizen scientists were unavailable for one or more samplings; finally, we did not actively involve special habitats for sampling. It has been shown that honey bees tend not to forage on all pollen producing plants in a habitat^15,46,47^. The low-abundance pollen types, detected through this study may partly originate from pollen sources accidentially collected by bees and can in some cases be regarded as external contamination like bee to bee contact or windblown dispersal of pollen grains^36,48,49^. One example for anemophilous plant taxons was reported by Vaissière and Vinson^49^: maize pollen with its powdery structure easily sticks to the haircoat of nearby bees and gets transported into the bee colony, though for maize pollen active collection is also possible^8,50^.

A certain underestimation in the total number of species found is further likely because of pollen forms not distinguishable by light microscopy, which sometimes does not allow to assign all pollen grains to their botanical origins or to species level^38,50^. Instead, they were allocated to genus or family level, or they were summarized as either ‘various unknown’ with or without coding or as cryptic groups. Even though there was a large number of specimens available in the database used in this investigation, the composite (heterogenous) group of ‘various unknown’ pollen forms was found to make up the highest occurring pollen type, but with low abundance. The majority of other important (and the highly abundant) pollen types could well be determined by light microscopy. The use of molecular genetic methods may also increase the number of identified pollen sources^50,51^. They are known to determine a greater number of plant species than classical palynological methods with different limitations^18,52–54^. For instance, Richardson *et al*.^51^ reported on identified genera that were implausible due to their floral morphology. However, in contrast to genetic identification of botanical origin, our palynological analysis allowed the calculation of abundance of pollen types by counting pollen grains and relating them to each other. Finally, citizen scientists could, compared to more elaborate methods for molecular analysis, easily preserve the specimens for microscopic analysis by drying and freezing pollen samples.

Nevertheless, within this study we demonstrate that honey bees in Austria forage on a broader pollen source spectrum compared to many studies conducted in other countries^15,21,25,27,28,32,38,46,55–59^. To cite only a few examples, a comparative sampling and analysis in Northwestern Italy found a total of 90 different ‘palynological types’^32^. At a single site, and over 9 sampling dates, the highest number of different pollen sources in our study was 105. The average of 18 complete sampling series per year was 84.5 (±9.0) per site. This can be compared to single sites investigated in other countries. For example in Ireland, 76 different pollen types were identified in the observational period of 1993–1994^27^, while in an intensively cultivated agro-ecosystem in North Dakota, from May to September 2014 (bi-)weekly pollen collection resulted in 114 different pollen sources^25^. Depending on the sampling and analysis methods used by the authors, up to 142 pollen types were reported over a two years observational period in Thessaloniki, Greece^15^. At the other extreme, Ismail *et al*. reported on only 24 pollen taxons at a single site in Egypt during two successive years^58^.

The pollen diversity presented in our study is also obvious when focusing on the sampling dates over the study period. While authors like Girard *et al*.^21^ found between 43 and 54 pollen types and Avni *et al*.^55^ reported on 5 to 20 pollen taxons, we were, regardless of the season, always able to identify a minimum of 62 different pollen taxa for each sampling date in Austria. Furthermore, the 85 plant families providing pollen to bees in Austria largely exceed the 39 families found in California by Lau *et al*.^26^. The comparatively high number of pollen species we found may either suggest a really large number of pollen sources in Austria, but might also come from methodological differences to the other studies. We ascribe the high number at least partly to our country-wide collection of a large number of samples in a lot of different locations compared to many single site studies.

Although 239 distinct pollen forms were recorded for both years, the majority of them was collected in quantities lower than 1% abundance, while dominant pollen sources with abundances higher than 20% were comparably rare (Fig. 6a). This suggests, that only a relatively small amount of analysed pollen types are important pollen sources for honey bee nutrition in Austria. From a practical point of view, these species should be conserved or planted preferably to improve bees’ pollen forage, instead of planting rare species of low attractiveness. It is possible that locally important species may be under-represented in our study because the abundance was not calculated based on samples on colony or location level but on the number of pollen grains for a specific sampling date (Fig. 6a). As a consequence, plant species of local importance may be under-represented in this more general approach. In other words, from the 120 pollen taxons with abundances lower than 1% found in early June, some may be highly accumulated within individual samples, and some may be only present in traces but rather were found in many samples. Hence, the presentation of the pollen types’ abundancies over the sampling date does not provide a reliable statement for individual bee colonies, but for the whole study population.

These findings are again comparable to other studies on pollen diversity^21,26,32^. Compared to the study in Thessaloniki, Greece^15^, we identified more pollen types, which can be attributed to the larger number of rare pollen types in Austria (Fig. 6a). According to Goulson^60^, young forager bees lack experience in recognizing rewarding pollen sources. Consequently, they may visit plants with little or no available pollen and thus unintentionally pick up a few pollen grains. Furthermore, it is possible that bees visiting flowers that have been visited shortly before by other foragers receive a lower rate of reward^60,61^. The significance of such rare pollen taxons or their contribution to a diverse diet in honey bee nutrition requires further research. Nevertheless, increasing the knowledge on pollen diversity demands learning about rarely appearing pollen sources too. To identify the most common plants that provide pollen to honey bees, sampling only a few honey bee colonies seems to be sufficient. To detect rare plant taxons, or plant species of local importance, more test apiaries are essential, especially in the context of monitoring plant biodiversity with honey bees. One possibility to investigate the latter might be to mix pollen samples from several colonies of the same apiary for analysis^23^.

Occurrence and abundance of the identified pollen types helps us to better understand the pollen source availability for honey bee nutrition. The occurrence describes which pollen types have been collected by bees, whereas the abundance better describes what pollen types bees feed on over the season. The latter suggests that bee colonies in Austria mostly feed on pollen from *Trifolium repens*, *Plantago* spp., *Salix* spp., pome fruit trees and *Taraxacum*-form (Fig. 3b). In Fig. 3, both are contrasted, which shows no major overlap between the most occurring and the most abundant species. It is remarkable, that the occurrence of the most important pollen sources does not differ greatly between the two study years even though some sampling locations have changed between the two study years (Fig. 1). Contrary, the abundance fluctuated more between the two years. We found that the top ten most abundant pollen types accounted for 48% of all pollen grains collected during the investigation period. To better understand the contribution of these abundant pollen types to bee nutrition, further research on some characteristics of these pollen types is needed. These include protein content, pollen grain size and pollen digestibility. For example, Liolios *et al*.^22^ demonstrated that only 14 pollen taxa significantly contribute to bee nutrition by providing almost 90% of a colony’s annual protein supply. Pollen grain size must also be taken into account to avoid an under- or overestimation of abundances when using classical (melisso-)palynological methods. Taking into account that the assessment of each pollen species’ volume is no easy task, it is considered to be more representative than standard grain counting^23,34,56,62^.

In spring, the most abundant pollen sources for honey bees were typical spring bloomers such as willow, pome fruit trees, maple, *Brassica* spp. and dandelion, while white clover and the plantain genus were present continuously from summer to autumn. The sampling date in September marked the end of the season with again plantain, together with ivy being the most abundant pollen sources. It has to be mentioned, that by comparing the top 10 pollen sources, there are differences between occurrence and abundance data. For example, we found *Salix* spp. to be the most abundant pollen source in spring as it was reported from Girard *et al*.^21^ for June and July in Canada. The low occurrence in our study suggests high availability of *Salix* spp. for a limited number of colonies. One explanation is that our spring sampling dates already missed the (earlier) willow bloom at lower altitudes. In autumn, we identified ivy (*Hedera helix*) to be a very abundant pollen source, confirming Garbuzov and Ratnieks^63^, but again occurrence data is not consistent. The major reason for this discrepancy is, that 10.7% of the 168 samples in September were identified to contain almost exclusively *H. helix* pollen, resulting in high abundance.

Assigning all identified pollen taxa to plant family level revealed that the known insect pollinated families Asteraceae, Fabaceae and Rosaceae were the three most important families providing pollen to honey bees in our study. This is consistent with findings in Greece^15^ and by Kirk^64^ who analysed bee collected pollen loads from England and Germany. The great importance of Asteraceae is not surprising, since this is the most species-rich taxonomic family in Austria including 467 botanical taxa. Rosaceae are ranked number three with 305 taxa and Fabaceae, with 138 taxa, is set on the sixth place in Austria^44^. While we found pollen from the family Asteraceae in 93.5% of all samples, pollen from Austria’s second largest plant family Poaceae (238 taxa) appeared in 46.9% samples, more often than previously thought in bee collected pollen samples^44^. However, to assess the contribution of wind pollinated Poaceae pollen to honey bee nutrition, the often very low abundance needs to be considered. Among the pollen of Poaceae, only two groups could be distinguished: a cryptic *Zea mays* and *Triticum aestivum* group and pollen belonging to the family Poaceae in general. The latter were found in high occurrence but low abundance, again indicating accidental collection or contamination^48^. However, in one case the cryptic group of *Z. mays* and *T. aestivum*, and in three cases the not further distinguishable Poaceae pollen were even found as dominant (>50%) contributor (see Table S3). Altough, *T. aestivum* was not distinguishable from *Z. mays* by light microscopy, we assume that the latter forms the monofloral pollen sample as pollen collection from maize has been reported before^8,50^. Maurizio and Schaper list maize with a pollen value of 4^42^, whereas the nutritional quality for bees is regarded low^65^. The three pollen samples dominated by Poaceae also indicate to active pollen collection by bees^66^.

Trees were found to be important pollen donors for honey bees throughout the season, but similar to Odoux *et al*.^23^, who studied one agricultural site in France, we found trees to be the most abundant pollen source in spring with 59.7% of analysed pollen grains. Later in the season, pollen from trees are replaced by pollen from herbs. In contrast  to the French study^23^, we did not find a peak of pollen from trees in late summer, but this is probably because we additionally differentiated for the growth-form climber/creeper, which for example includes *H. helix* and *Parthenocissus* spp., two important late summer pollen sources. Our findings underline the importance of trees for pollinator nutrition, especially in spring. The reasons for the high abundance of pollen from woods in spring are manifold and include the trees’ ability to produce more flowers and therefore more pollen^23^.

A pollen sample on average consisted of 15.0 to 16.8 pollen types in 2014 and 2015, respectively. We found a seasonal pattern for the average number of pollen types collected per colony. While at the beginning (early April) and the end of the season (September) the mean numbers of different pollen types per colony were low, the highest number of pollen types was collected by bees in May followed by June (Fig. 5). This pattern is also visible in the total number of pollen types found at each sampling date – from 92 recorded in early April up to 145 in early June (Fig. 6a), though in this part of the analysis the lower number at the beginning and at the end of the season could be due to the failure in sampling the required amount needed for analysis, as seen by lower number of colonies and locations sampled (Table 1). Similar investigations demonstrating seasonal variation in pollen diversity with peaks in spring and summer already exist for France^23^. However their results stem from only 4 sampling locations. In Ireland, seasonal differences of pollen availability with the diversity of species being foraged reaching a maximum during June and July were reported^27^.

Simpson’s diversity index further highlights this seasonal pattern (Fig. 7). The highest median diversity (0.756) was found in May, whereas it was lower earlier in the year and least in September (0.515). The median diversity index over the whole season was 0.668. This is higher than the diversity indices reported from Tennessee^19^ ranging from 0.106 to 0.176. Their methods are widely comparable to ours with the exception that they investigated agricultural and non-agricultural areas. Still, the large discrepancy between the two studies could also be due to different calculation of indices. Studies on pollen diversity including the Simpson’s index, especially in the European area, are rare. Therefore, it should be considered to calculate the Simpson’s diversity index in further palynological studies on pollen diversity to improve comparability of results. Figure 7 also depicts the large variations in the diversity of pollen samples within one sampling date. These variations most likely come from a different availability of pollen in different ecological habitats^17,19,25,28^, or in spring from decelerated development and late blooming in elevated mountainous sampling locations. As floral availability in the environment affects colony development and health status of bees^39^, the reasons for the geographic variations in pollen diversity and connection to landscape composition in Austria deserve further analysis in a separate article.

Our study demonstrated that half of all samples were to a great part (>50%) dominated by pollen from one botanical source, 4.2% contained more than 90% of one pollen type. Such high dominances by single pollen sources have sporadically been reported before^8,55,63^. The impacts of such one-sided pollen diets on honey bee health have been studied in the laboratory using monofloral pollen diets^7–10^. Compared to mixed diets, monofloral pollen diets are often, but not always, deemed to be of lower nutritional value to honey bees. To our knowledge, our study provides one of the most comprehensive and systematic reports of the natural occurrence of pollen diets widely comprised of the pollen of one species. The pollen types we identified to dominate naturally occurring nearly monofloral samples are from wild plants as well as from agricultural crops (Tables 2 and S3). Though the protein content of those pollen is well investigated^67^, further research on the suitability for honey bees of some of the monofloral pollen diets we identified is needed. Omar *et al*.^9^ investigated two of the pollen types making up nearly monofloral pollen samples in our study. They found that the pollen from *Sinapis* spp., often grown as a catch crop, is of poor quality for honey bees (according to Maurizio and Schaper^42^ *Sinapis* is rated with a 2 for nectar and pollen value) compared to other monofloral diets or a mixed diet, whereas the monofloral pollen of *Castanea sativa* is more nutritive (rated with a 4 for nectar and 3 for the pollen value^42^). Di Pasquale *et al*.^7^ attested a monofloral *Rubus* spp. diet, also sometimes found naturally occurring nearly monofloral in our study, the same nutritional quality as that of a mixed pollen diet in their experiments.

The seasonal pattern as demonstrated by number of pollen species and Simpson’s diversity index is inversely reflected in the relative frequency of the nearly monofloral pollen samples (Fig. 6b). Such samples were less frequently found in May, more often in April and most frequent in September. In autumn we detected some samples almost exclusively comprised of *H. helix* (Fig. 7c). Ivy is recognized as an important source of pollen (and nectar) in autumn^63^. At the end of the season, ivy provides the diet of which long lived winter bees are made of and the stored pollen is used to commence brood rearing in the following season. Nevertheless, colonies collected pollen samples highly dominated by one pollen type also in June and July. Whereas very likely shortages in pollen source diversity underlie the nearly monofloral pollen samples in spring and autumn, further research is needed whether there are other reasons, such as high availability or good nutritional quality, for some colonies almost exclusively collecting pollen from *C. sativa*, *Plantago* spp., *Rubus* spp. and *Trifolium pratense*.

For the first time we conducted a nationwide investigation on pollen diversity available to honey bee colonies in Austria. The large area covered and high number of samples obtained were only possible due to the combination of citizen scientist sampling and expert microscopy pollen analysis. We found a high total floral diversity of pollen sources – 239 pollen species from 85 families, with a mean number of 16.2 species in one sample and a median Simpson’s diversity index of 0.668. The composition of pollen samples was highly variable ranging from diverse diets to about half of all samples being dominated by pollen from a single species particularly in spring and late summer/autumn. Additionally, our results hint, next to low pollen diversity in spring and autumn, to other possible reasons for monofloral pollen samples, which require further research. Investigations on the pollen provisioning quality of different landscapes for honey bee colony health and welfare, termed ‘landscape physiology’ by Alaux *et al*.^6^, remain a topic that can facilitate the understanding of honey bee foraging ecology and colony health.

## Supplementary information

Supplementary tables

## Data Availability

All summary data of pollen analysis (occurrence and abundance of all species) are available in supplementary material. The GPS data regarding the sample site locations are not publicly available to preserve the privacy of participating citizen scientists. Individual data are however available from the authors upon reasonable request.
