# Supplementary material for: A citizen science supported study on seasonal diversity and monoflorality of pollen collected by honey bees in Austria
Source: Sci Rep. 2019 Nov 12;9:16633. doi: 10.1038/s41598-019-53016-5 (PMC6851371; doi:10.1038/s41598-019-53016-5)
Supplement: Supplementary file 1 — Supplementary tables [file 41598_2019_53016_MOESM1_ESM.pdf]

Supplementary Information

A citizen science supported study on seasonal diversity and monoflorality of pollen collected by honey bees in Austria

Robert Brodschneider[1]*, Kristina Gratzer[1], Elfriede Kalcher-Sommersguter[1], Helmut Heigl[2], Waltraud Auer[2], Rudolf Moosbeckhofer[2], Karl Crailsheim[1]

1 University of Graz, Institute of Biology, Universitätsplatz 2, 8010 Graz, Austria

2 Austrian Agency for Health and Food Safety, Ltd., Institute for Seed and Propagating Material, Plant Protection Service and Apiculture, Department for Apiculture and Bee Protection, Vienna, Austria

* corresponding author email: robert.brodschneider@uni-graz.at

List of Supplementary Tables

Supplementary Table S1: Occurrence of all 239 pollen types and their growth-form for 2014, 2015 and for both years combined in alphabetical order. Occurrence of positively tested pollen samples and its proportional frequency [%] is based on a sample size of n=804 in 2014, n=818 in 2015 and n=1622 for both years combined. The maximum contribution in a sample is based on ~500 analysed pollen grains per positively tested sample. Explanatory notes are found at the end of the table.

Supplementary Table S2: Abundance of all 239 pollen forms [%] identified in 2014, 2015 and for both years combined in alphabetical order. The relative abundance depends on the total number of analysed pollen grains: n=401603; n=409001; n=810604 for 2014, 2015 and for both years, respectively. Explanatory notes are found at the end of the table.

Supplementary Table S3: Number and frequency of partially (>50% comprised of one pollen type) and highly (>90%) monofloral pollen samples collected in Austria in 2014 and 2015 combined (n=1622 pollen samples). *Form cryptic groups, not further differentiable by light microscopy.

Table S1: Occurrence of all 239 pollen types and their growth-form for 2014, 2015 and for both years combined in alphabetical order. Occurrence of positively tested pollen samples and its proportional frequency [%] is based on a sample size of n=804 in 2014, n=818 in 2015 and n=1622 for both years combined. The maximum contribution in a sample is based on ~500 analysed pollen grains per positively tested sample. Explanatory notes are found at the end of the table.

		**2014**			**2015**			**Both years**		
**Species**	**Growth-form**	**Occurrence of positively tested pollen samples**	**% of positively tested pollen samples**	**Maximum contribution of pollen type in a sample [%]**	**Occurrence of positively tested pollen samples**	**% of positively tested pollen samples**	**Maximum contribution of pollen type in a sample [%]**	**Occurrence of positively tested pollen samples**	**% of positively tested pollen samples**	**Maximum contribution of pollen type in a sample [%]**
Acer spp.	Tree	256	31.8	87.2	183	22.4	91.2	439	27.1	91.2
Achillea-form[1]	Herb	271	33.7	63.4	187	22.9	51.6	458	28.2	63.4
Aesculus hippocastanum	Tree	129	16.0	77.0	105	12.8	45.8	234	14.4	77.0
Aesculus x carnea	Tree	31	3.9	31.2	18	2.2	45.4	49	3	45.4
Ailanthus altissima	Tree	17	2.1	3.0	22	2.7	24.6	39	2.4	24.6
Ajuga spp.	Herb	5	0.6	13.0	0	0.0	0.0	5	0.3	13.0
Allium spp.	Herb	77	9.6	3.2	78	9.5	6.0	155	9.6	6.0
Alnus spp.	Tree	8	1.0	0.6	8	1.0	0.4	16	1	0.6
Ambrosia spp.	Herb	5	0.6	40.0	8	1.0	9.4	13	0.8	40.0
Amorpha fruticosa	Shrub	103	12.8	28.4	85	10.4	54.6	188	11.6	54.6
Anemone spp., *Clematis* spp., *Pulsatilla* spp., *Ranunculus* spp.*	UGF**	2	0.2	12.4	418	51.1	65.0	420	25.9	65.0
Apiaceae	UGF**	296	36.8	70.8	247	30.2	88.8	543	33.5	88.8
Arctium spp.	Herb	53	6.6	16.4	52	6.4	6.8	105	6.5	16.4
Artemisia spp.	Herb	25	3.1	12.2	26	3.2	17.6	51	3.1	17.6
Aruncus dioicus	Herb	69	8.6	38.4	46	5.6	32.0	115	7.1	38.4
Asparagus officinalis	Herb	17	2.1	61.4	14	1.7	20.0	31	1.9	61.4
Asperula spp., *Cruciata* spp., *Galium* spp.*	Herb	19	2.4	12.4	7	0.9	0.8	26	1.6	12.4

Asteraceae	UGF**	408	50.7	83.6	407	49.8	82.4	815	50.2	83.6
Atriplex spp., *Bassia* spp., *Chenopodium* spp., *Suaeda* spp.*	UGF**	100	12.4	16.0	109	13.3	60.2	209	12.9	60.2
Atropa bella-donna	Herb	31	3.9	2.8	14	1.7	1.2	45	2.8	2.8
Begonia spp.	Herb	62	7.7	78.6	50	6.1	46.0	112	6.9	78.6
Berberidaceae	UGF**	51	6.3	4.8	60	7.3	8.8	111	6.8	8.8
Betula spp.	Tree	71	8.8	58.2	29	3.5	40.8	100	6.2	58.2
Boraginaceae	UGF**	1	0.1	1.6	2	0.2	0.2	3	0.2	1.6
Borago officinalis	Herb	6	0.7	1.2	1	0.1	8.6	7	0.4	8.6
Brassica spp.	Herb	95	11.8	79.0	80	9.8	82.2	175	10.8	82.2
Brassicaceae	UGF**	339	42.2	80.0	375	45.8	79.4	714	44	80.0
Buddleja spp., *Cyclamen* spp., *Primula* spp.*	UGF**	136	16.9	65.4	119	14.5	66.6	255	15.7	66.6
Buxus sempervirens	Shrub	28	3.5	19.6	56	6.8	22.2	84	5.2	22.2
Calluna vulgaris	Shrub	14	1.7	26.6	42	5.1	64.4	56	3.5	64.4
Calystegia spp.	Climber/ creeper	114	14.2	0.8	85	10.4	1.6	199	12.3	1.6
Campanula spp., *Jasione* spp., *Legousia* spp., *Phyteuma* spp.*	Herb	72	9.0	2.8	42	5.1	4.6	114	7	4.6
Cannabis sativa	Herb	1	0.1	0.2	11	1.3	70.6	12	0.7	70.6
Carpinus betulus	Tree	38	4.7	6.2	18	2.2	1.0	56	3.5	6.2
Caryophyllaceae	UGF**	194	24.1	5.4	133	16.3	10.0	327	20.2	10.0
Castanea sativa	Tree	51	6.3	92.8	55	6.7	91.6	106	6.5	92.8
Catalpa spp.	Tree	4	0.5	0.2	3	0.4	0.2	7	0.4	0.2
Centaurea jacea-form	Herb	211	26.2	46.8	150	18.3	26.2	361	22.3	46.8

Centaurea scabiosa	Herb	3	0.4	1.6	4	0.5	0.4	7	0.4	1.6
Cercis siliquastrum	Tree	4	0.5	7.8	0	0.0	0.0	4	0.2	7.8
Cirsium spp., *Carduus* spp., *Silybum* spp.*	Herb	317	39.4	35.0	320	39.1	48.6	637	39.3	48.6
Cistus spp.	Shrub	0	0.0	0.0	1	0.1	0.2	1	0.1	0.2
Citrus spp.	Tree	3	0.4	1.4	0	0.0	0.0	3	0.2	1.4
Clinopodium vulgare	Herb	0	0.0	0.0	1	0.1	0.2	1	0.1	0.2
Colchicum autumnale	Herb	23	2.9	1.2	13	1.6	2.8	36	2.2	2.8
Colchicum spp.	Herb	19	2.4	1.4	22	2.7	2.0	41	2.5	2.0
Convolvulus spp.	Herb	132	16.4	3.4	105	12.8	24.8	237	14.6	24.8
Cornus sanguinea	Shrub	155	19.3	20.6	127	15.5	37.6	282	17.4	37.6
Corylus avellana	Shrub	14	1.7	9.8	2	0.2	0.2	16	1	9.8
Cotinus coggygria	Shrub	9	1.1	46.8	4	0.5	54.6	13	0.8	54.6
Cotoneaster spp.	Shrub	3	0.4	30.8	0	0.0	0.0	3	0.2	30.8
Crocus spp.	Herb	25	3.1	3.0	13	1.6	1.4	38	2.3	3.0
Cucurbita pepo	Climber/ creeper	16	2.0	0.4	14	1.7	0.2	30	1.8	0.4
Cucurbitaceae	UGF**	4	0.5	0.8	13	1.6	1.0	17	1	1.0
Cyanus montanus	Herb	1	0.1	0.2	1	0.1	0.4	2	0.1	0.4
Cyanus segetum	Herb	98	12.2	60.0	78	9.5	18.0	176	10.9	60.0
Cyanus triumfetti	Herb	0	0.0	0.0	26	3.2	24.0	26	1.6	24.0
Cyperaceae	UGF**	79	9.8	4.2	72	8.8	4.4	151	9.3	4.4
Datura spp.	Herb	3	0.4	0.4	4	0.5	1.6	7	0.4	1.6
Datura stramonium	Herb	1	0.1	0.2	5	0.6	0.4	6	0.4	0.4
Datura suaveolens	Shrub	1	0.1	0.2	1	0.1	2.0	2	0.1	2.0

Diervilla spp.	Shrub	10	1.2	0.4	13	1.6	10.6	23	1.4	10.6
Echinops sphaerocephalus	Herb	30	3.7	6.4	13	1.6	1.2	43	2.7	6.4
Echium spp.	Herb	54	6.7	36.0	37	4.5	10.4	91	5.6	36.0
Elaeagnus angustifolia	Tree	8	1.0	1.0	22	2.7	10.0	30	1.8	10.0
Epilobium angustifolium	Herb	20	2.5	0.4	17	2.1	0.8	37	2.3	0.8
Epilobium spp.	Herb	15	1.9	1.0	26	3.2	2.0	41	2.5	2.0
Erica arborea	Shrub	1	0.1	0.2	0	0.0	0.0	1	0.1	0.2
Erica carnea	Shrub	19	2.4	4.0	17	2.1	5.2	36	2.2	5.2
Ericaceae	UGF**	10	1.2	0.4	42	5.1	11.6	52	3.2	11.6
Euphorbia spp.	Herb	3	0.4	3.2	4	0.5	6.0	7	0.4	6.0
Fagopyrum esculentum	Herb	105	13.1	83.0	154	18.8	80.4	259	16	83.0
Fagus sylvatica	Tree	72	9.0	28.6	28	3.4	1.2	100	6.2	28.6
Filipendula spp.	Herb	89	11.1	79.6	71	8.7	75.0	160	9.9	79.6
Fragaria ananassa	Herb	13	1.6	19.8	14	1.7	19.0	27	1.7	19.8
Fragaria spp.	Herb	0	0.0	0.0	3	0.4	6.6	3	0.2	6.6
Fragaria vesca	Herb	60	7.5	14.6	27	3.3	11.0	87	5.4	14.6
Frangula alnus	Shrub	18	2.2	1.0	27	3.3	7.4	45	2.8	7.4
Fraxinus excelsior	Tree	17	2.1	3.6	39	4.8	29.6	56	3.5	29.6
Fraxinus ornus	Tree	0	0.0	0.0	14	1.7	21.4	14	0.9	21.4
Genista spp.	Herb	3	0.4	1.2	0	0.0	0.0	3	0.2	1.2
Geranium spp.	Herb	210	26.1	6.4	159	19.4	1.6	369	22.7	6.4
Geum spp.	Herb	1	0.1	5.2	0	0.0	0.0	1	0.1	5.2
Gleditsia triacanthos	Tree	36	4.5	79.6	62	7.6	46.4	98	6	79.6
Hedera helix	Climber/ creeper	104	12.9	99.8	75	9.2	99.8	179	11	99.8
Helianthemum spp.	Herb	128	15.9	8.2	114	13.9	33.8	242	14.9	33.8
Helianthus spp.	Herb	95	11.8	36.8	80	9.8	44.4	175	10.8	44.4

Helleborus niger	Herb	6	0.7	5.0	0	0.0	0.0	6	0.4	5.0
Helleborus spp.	Herb	0	0.0	0.0	10	1.2	5.2	10	0.6	5.2
Heracleum spp.	Herb	106	13.2	79.6	83	10.1	27.8	189	11.7	79.6
Hibiscus spp.	Shrub	3	0.4	0.2	2	0.2	0.2	5	0.3	0.2
Hippocrepis spp.	Herb	1	0.1	4.8	5	0.6	49.4	6	0.4	49.4
Humulus lupulus	Climber/ creeper	0	0.0	0.0	2	0.2	7.0	2	0.1	7.0
Hyacinthaceae	UGF**	4	0.5	0.4	20	2.4	0.6	24	1.5	0.6
Hydrangea spp.	Herb	12	1.5	42.8	26	3.2	85.2	38	2.3	85.2
Hypericum spp.	Herb	31	3.9	72.2	39	4.8	92.4	70	4.3	92.4
Ilex aquifolium	Tree	16	2.0	11.8	19	2.3	4.0	35	2.2	11.8
Impatiens spp.	Herb	178	22.1	98.2	151	18.5	93.6	329	20.3	98.2
Iridaceae	UGF**	1	0.1	0.2	1	0.1	0.2	2	0.1	0.2
Juglans spp.	Tree	77	9.6	9.2	64	7.8	13.2	141	8.7	13.2
Juncus spp., *Luzula* spp.*	Herb	77	9.6	31.8	57	7.0	43.8	134	8.3	43.8
Juniperus communis	Shrub	4	0.5	2.6	2	0.2	1.2	6	0.4	2.6
Knautia spp.	Herb	183	22.8	9.6	114	13.9	1.2	297	18.3	9.6
Lamiaceae	UGF**	1	0.1	2.0	0	0.0	0.0	1	0.1	2.0
Lamiaceae-form[2] *(6 Kst.)*	Herb	156	19.4	4.4	142	17.4	10.0	298	18.4	10.0
Lamiaceae-form[3] *(3 Kst.)*	Herb	79	9.8	46.4	48	5.9	17.2	127	7.8	46.4
Lantana camara	Shrub	0	0.0	0.0	2	0.2	0.2	2	0.1	0.2
Lathyrus spp.	Herb	4	0.5	1.4	3	0.4	3.2	7	0.4	3.2
Leucojum vernum	Herb	2	0.2	3.0	0	0.0	0.0	2	0.1	3.0
Ligustrum vulgare	Shrub	98	12.2	48.6	105	12.8	73.8	203	12.5	73.8
Lilium spp.	Herb	94	11.7	4.8	75	9.2	6.8	169	10.4	6.8
Linum spp.	Herb	6	0.7	0.6	3	0.4	0.2	9	0.6	0.6
Liriodendron tulipifera	Tree	50	6.2	16.4	36	4.4	43.6	86	5.3	43.6
Lonicera spp.	Shrub	116	14.4	5.4	78	9.5	11.2	194	12	11.2

Loranthus europaeus	Climber/ creeper	12	1.5	63.4	10	1.2	10.2	22	1.4	63.4
Lotus spp.	Herb	90	11.2	23.0	63	7.7	22.8	153	9.4	23.0
Lunaria rediviva	Herb	0	0.0	0.0	3	0.4	10.4	3	0.2	10.4
Lupinus spp.	Herb	3	0.4	6.6	0	0.0	0.0	3	0.2	6.6
Lysimachia spp.	Herb	0	0.0	0.0	79	9.7	15.8	79	4.9	15.8
Lythrum salicaria	Herb	27	3.4	12.8	47	5.7	86.0	74	4.6	86.0
Macleaya cordata	Herb	0	0.0	0.0	1	0.1	1.4	1	0.1	1.4
Malus spp., *Pyrus* spp., *Crataegus* spp.*	Tree	246	30.6	71.4	199	24.3	79.0	445	27.4	79.0
Malva-form[4]	Herb	44	5.5	0.6	32	3.9	0.4	76	4.7	0.6
Medicago spp.	Herb	0	0.0	0.0	5	0.6	54.2	5	0.3	54.2
Melampyrum spp.	Herb	22	2.7	78.4	35	4.3	90.0	57	3.5	90.0
Mercurialis spp.	Herb	4	0.5	9.6	0	0.0	0.0	4	0.2	9.6
Muscari spp.	Herb	22	2.7	6.8	18	2.2	1.8	40	2.5	6.8
Myosotis spp.	Herb	27	3.4	6.0	21	2.6	21.2	48	3	21.2
Narcissus spp.	Herb	3	0.4	1.8	0	0.0	0.0	3	0.2	1.8
not swollen	UGF**	36	4.5	73.8	23	2.8	40.6	59	3.6	73.8
Nymphaea spp.	Herb	4	0.5	1.0	2	0.2	0.2	6	0.4	1.0
Ocimum basilicum	Herb	26	3.2	1.0	44	5.4	0.6	70	4.3	1.0
Oenothera tetragona	Herb	63	7.8	1.6	60	7.3	0.4	123	7.6	1.6
Onobrychis spp.	Herb	50	6.2	27.8	22	2.7	26.4	72	4.4	27.8
Ononis spinosa	Herb	4	0.5	5.6	0	0.0	0.0	4	0.2	5.6
Onopordum spp.	Herb	1	0.1	0.2	1	0.1	0.2	2	0.1	0.2
Ornithogalum spp.	Herb	3	0.4	6.4	0	0.0	0.0	3	0.2	6.4
Pachysandra terminalis	Herb	3	0.4	0.2	3	0.4	0.4	6	0.4	0.4
Paeonia spp.	Shrub	5	0.6	0.2	0	0.0	0.0	5	0.3	0.2

Papaver rhoeas	Herb	45	5.6	79.2	16	2.0	57.2	61	3.8	79.2
Papaver somniferum	Herb	5	0.6	79.6	6	0.7	43.6	11	0.7	79.6
Papaver spp.	Herb	4	0.5	31.0	6	0.7	56.2	10	0.6	56.2
Parthenocissus spp.	Climber/ creeper	185	23.0	74.0	214	26.2	86.0	399	24.6	86.0
Passiflora spp.	Herb	0	0.0	0.0	3	0.4	0.2	3	0.2	0.2
Persicaria bistorta	Herb	18	2.2	4.4	17	2.1	0.6	35	2.2	4.4
Persicaria maculosa	Herb	1	0.1	0.2	23	2.8	1.6	24	1.5	1.6
Petasites spp.	Herb	23	2.9	14.4	19	2.3	3.4	42	2.6	14.4
Petunia spp.	Shrub	49	6.1	38.8	70	8.6	59.4	119	7.3	59.4
Phacelia tanacetifolia	Herb	52	6.5	64.6	74	9.0	87.4	126	7.8	87.4
Phlox spp.	Herb	5	0.6	0.4	1	0.1	0.2	6	0.4	0.4
Physocarpus monogynus	Shrub	5	0.6	44.0	14	1.7	88.4	19	1.2	88.4
Picea abies, Abies alba	Tree	128	15.9	0.6	142	17.4	1.0	270	16.6	1.0
Pinus spp.	Tree	142	17.7	65.4	156	19.1	4.2	298	18.4	65.4
Plantago spp.	Herb	541	67.3	72.8	567	69.3	99.0	1108	68.3	99.0
Platanus acerifolia	Tree	0	0.0	0.0	11	1.3	4.0	11	0.7	4.0
Poaceae	UGF**	328	40.8	32.0	329	40.2	59.0	657	40.5	59.0
Polemonium caeruleum	Herb	5	0.6	0.6	6	0.7	0.2	11	0.7	0.6
Populus spp.	Tree	0	0.0	0.0	3	0.4	2.8	3	0.2	2.8
Potentilla spp.	Herb	101	12.6	79.6	65	7.9	28.2	166	10.2	79.6
Primula acaulis, P. elatior, P. veris	Herb	0	0.0	0.0	5	0.6	0.8	5	0.3	0.8
Prunus avium	Tree	22	2.7	70.4	0	0.0	0.0	22	1.4	70.4
Prunus domestica	Tree	12	1.5	8.4	51	6.2	5.8	63	3.9	8.4
Prunus padus	Tree	33	4.1	17.4	9	1.1	64.4	42	2.6	64.4

Prunus persica	Tree	1	0.1	0.8	0	0.0	0.0	1	0.1	0.8
Prunus spp.	Tree	87	10.8	71.2	131	16.0	83.4	218	13.4	83.4
Pterocarya fraxinifolia	Tree	3	0.4	1.2	3	0.4	7.2	6	0.4	7.2
Pulmonaria spp.	Herb	0	0.0	0.0	1	0.1	0.4	1	0.1	0.4
Quercus spp.	Tree	78	9.7	45.0	27	3.3	22.2	105	6.5	45.0
Ranunculus spp.	Herb	495	61.6	86.8	0	0.0	0.0	495	30.5	86.8
Rhamnus spp.	Shrub	35	4.4	35.0	26	3.2	32.8	61	3.8	35.0
Rhododendron spp., *Vaccinium* spp.*	Shrub	50	6.2	18.2	18	2.2	12.2	68	4.2	18.2
Ribes spp.	Shrub	7	0.9	2.8	0	0.0	0.0	7	0.4	2.8
Robinia pseudacacia	Tree	15	1.9	28.2	36	4.4	88.2	51	3.1	88.2
Rosaceae	UGF**	155	19.3	38.4	102	12.5	39.2	257	15.8	39.2
Rubiaceae	UGF**	0	0.0	0.0	5	0.6	8.2	5	0.3	8.2
Rubus spp.	Shrub	207	25.7	99.0	217	26.5	93.8	424	26.1	99.0
Rumex spp.	Herb	109	13.6	26.8	86	10.5	9.6	195	12	26.8
Salix spp.	Tree	202	25.1	92.2	151	18.5	99.8	353	21.8	99.8
Salvia glutinosa	Herb	75	9.3	88.6	65	7.9	91.6	140	8.6	91.6
Salvia officinalis	Herb	2	0.2	0.8	0	0.0	0.0	2	0.1	0.8
Sambucus nigra	Shrub	0	0.0	0.0	1	0.1	13.2	1	0.1	13.2
Sambucus spp., *Philadelphus coronarius**	Shrub	8	1.0	72.8	8	1.0	14.4	16	1	72.8
Sanguisorba officinalis	Herb	0	0.0	0.0	1	0.1	0.2	1	0.1	0.2
Scabiosa spp.	Herb	36	4.5	1.0	19	2.3	0.6	55	3.4	1.0
Scilla spp.	Herb	1	0.1	0.4	3	0.4	3.2	4	0.2	3.2
Sedum spp.	Herb	0	0.0	0.0	1	0.1	0.2	1	0.1	0.2
Sida hermaphrodita	Herb	0	0.0	0.0	5	0.6	1.8	5	0.3	1.8
Silphium perfoliatum	Herb	6	0.7	1.6	0	0.0	0.0	6	0.4	1.6
Sinapis spp.	Herb	186	23.1	91.0	114	13.9	99.2	300	18.5	99.2

Solanum spp.	Herb	0	0.0	0.0	4	0.5	13.8	4	0.2	13.8
Soldanella spp.	Herb	0	0.0	0.0	3	0.4	25.6	3	0.2	25.6
Sophora spp., *Linaria* spp.*	UGF**	1	0.1	0.2	0	0.0	0.0	1	0.1	0.2
Symphytum spp.	Herb	13	1.6	1.4	6	0.7	0.8	19	1.2	1.4
Syringa vulgaris	Shrub	110	13.7	45.4	86	10.5	33.2	196	12.1	45.4
Taraxacum-form[5]	Herb	606	75.4	79.8	598	73.1	76.0	1204	74.2	79.8
Taxus baccata	Tree	1	0.1	0.4	3	0.4	0.8	4	0.2	0.8
Thuja spp.	Tree	0	0.0	0.0	4	0.5	0.6	4	0.2	0.6
Tilia spp.	Tree	134	16.7	68.6	103	12.6	47.8	237	14.6	68.6
Tragopogon spp.	Herb	6	0.7	2.0	3	0.4	2.4	9	0.6	2.4
Trifolium pratense-form[6]	Herb	304	37.8	94.2	299	36.6	65.6	603	37.2	94.2
Trifolium repens-form[7]	Herb	502	62.4	87.4	499	61.0	93.6	1001	61.7	93.6
Ulmus spp.	Tree	0	0.0	0.0	1	0.1	14.8	1	0.1	14.8
unknown	UGF**	655	81.5	34.2	654	80.0	18.0	1309	80.7	34.2
unknown 223311	UGF**	0	0.0	0.0	9	1.1	32.4	9	0.6	32.4
unknown 223321	UGF**	0	0.0	0.0	1	0.1	0.2	1	0.1	0.2
unknown 223351	UGF**	3	0.4	11.6	2	0.2	27.4	5	0.3	27.4
unknown 223361	UGF**	34	4.2	50.8	30	3.7	36.0	64	3.9	50.8
unknown 333311	UGF**	2	0.2	58.2	5	0.6	26.2	7	0.4	58.2
unknown 333361	UGF**	11	1.4	34.0	29	3.5	61.6	40	2.5	61.6
unknown 334311	UGF**	6	0.7	14.2	0	0.0	0.0	6	0.4	14.2
unknown 443111	UGF**	4	0.5	20.6	0	0.0	0.0	4	0.2	20.6
unknown 443221	UGF**	7	0.9	75.2	0	0.0	0.0	7	0.4	75.2

unknown 443251	UGF**	0	0.0	0.0	4	0.5	41.2	4	0.2	41.2
unknown 443261	UGF**	2	0.2	3.2	4	0.5	85.0	6	0.4	85.0
unknown 443311	UGF**	0	0.0	0.0	12	1.5	32.6	12	0.7	32.6
unknown 443321	UGF**	7	0.9	15.4	3	0.4	16.2	10	0.6	16.2
unknown 443361	UGF**	9	1.1	47.4	7	0.9	13.0	16	1	47.4
unknown 553251	UGF**	0	0.0	0.0	4	0.5	13.0	4	0.2	13.0
unknown 553261	UGF**	16	2.0	24.6	0	0.0	0.0	16	1	24.6
unknown 553311	UGF**	1	0.1	48.8	0	0.0	0.0	1	0.1	48.8
unknown 553321	UGF**	0	0.0	0.0	1	0.1	10.6	1	0.1	10.6
unknown 553361	UGF**	2	0.2	20.2	7	0.9	10.0	9	0.6	20.2
unknown 663261	UGF**	3	0.4	39.6	0	0.0	0.0	3	0.2	39.6
unknown 663311	UGF**	0	0.0	0.0	1	0.1	15.0	1	0.1	15.0
unknown 663361	UGF**	2	0.2	4.4	2	0.2	16.4	4	0.2	16.4
unknown 773361	UGF**	1	0.1	6.0	0	0.0	0.0	1	0.1	6.0
unknown 776131	UGF**	1	0.1	5.8	0	0.0	0.0	1	0.1	5.8
Urtica spp., *Parietaria* spp.	Herb	13	1.6	12.6	3	0.4	0.6	16	1	12.6
Valeriana spp.	Herb	0	0.0	0.0	8	1.0	5.0	8	0.5	5.0
Verbascum spp.	Herb	0	0.0	0.0	5	0.6	28.6	5	0.3	28.6
Veronica spp.	Herb	3	0.4	4.2	0	0.0	0.0	3	0.2	4.2
Viburnum spp.	Shrub	69	8.6	49.0	60	7.3	16.6	129	8	49.0
Vicia spp.	Herb	107	13.3	84.2	59	7.2	98.8	166	10.2	98.8
Vinca spp.	Herb	0	0.0	0.0	1	0.1	0.2	1	0.1	0.2

Viola tricolor	Herb	97	12.1	3.0	68	8.3	1.2	165	10.2	3.0
Viscum album	Climber/ creeper	4	0.5	0.6	14	1.7	3.4	18	1.1	3.4
Vitis vinifera	Climber/ creeper	2	0.2	2.2	9	1.1	68.8	11	0.7	68.8
Weigela spp.	Shrub	39	4.9	1.8	25	3.1	1.2	64	3.9	1.8
Zea mays, Triticum aestivum	Herb	102	12.7	56.0	93	11.4	42.8	195	12	56.0

[1] *Achillea* spp., *Anthemis* spp., *Argyranthemum* spp., *Chrysanthemum* spp., *Leucanthemopsis* spp., *Leucanthemum* spp., *Tanacetum* spp., *Tripleurospermum* spp.

[2] *Acinos* spp., *Agastache* spp., *Clinopodium* spp., *Dracocephalum* spp., *Glechoma* spp., Lavandula spp., *Melissa* spp., *Mentha* spp., *Monarda* spp., *Nepeta* spp., *Ocimum* spp., *Origanum* spp., *Prunella* spp., *Rosmarinus* spp., *Salvia* spp., *Satureja* spp., *Thymus* spp.

[3] *Ajuga* spp., *Ballota* spp., *Betonica* spp., *Caryopteris* spp., *Galeobdolon* spp., *Galeopsis* spp., *Lamium* spp., *Leonorus* spp., *Marrubium* spp., *Melittis* spp., *Moluccella* spp., *Nepeta* spp., *Phlomis* spp., *Prasium* spp., *Scutellaria* spp., *Sideitis* spp., *Stachys* spp., *Teucrium* spp., *Vitex* spp.

[4] *Althaea* spp., *Dinacrusa hirsuta*, *Lavatera* spp., *Malva* spp.

[5] *Cicerbita* spp., *Cichorium* spp., *Crepis* spp., *Hieracium* spp., *Hypochaeris* spp., *Lactuca* spp., *Lapsana* spp., *Leontodon* spp., *Picris* spp., *Prenanthes* spp., *Reichardia* spp., *Scorzonera* spp., *Scorzoneroides* spp., *Sonchus* spp., *Taraxacum* spp., *Urospermum* spp., *Willemetia* spp.

[6] *T. pratense*, *T. hybridum*, *T. alexandrinum*, *T. incarnatum*

[7] *T. repens*, *T. montanum*, *T. nigrescens*

* Not further differentiable by light microscopy

** Unknown growth-form

Table S2: Abundance of all 239 pollen forms [%] identified in 2014, 2015 and for both years combined in alphabetical order. The relative abundance depends on the total number of analysed pollen grains: n=401603; n=409001; n=810604 for 2014, 2015 and for both years, respectively. Explanatory notes are found at the end of the table.

**Species**	**Abundance 2014 [%]**	**Abundance 2015 [%]**	**Abundance both years [%]**
Acer spp.	5.1	2.7	3.9
Achillea-form[1]	0.8	0.7	0.8
Aesculus hippocastanum	1.5	0.7	1.1
Aesculus x carnea	0.2	0.1	0.1
Ailanthus altissima	0.0	0.1	0.1
Ajuga spp.	0.0	0.0	0.0
Allium spp.	0.1	0.1	0.1
Alnus spp.	0.0	0.0	0.0
Ambrosia spp.	0.1	0.0	0.1
Amorpha fruticosa	0.2	0.3	0.3
Anemone spp., *Clematis* spp., *Pulsatilla* spp., *Ranunculus* spp.*	0.0	2.0	1.0
Apiaceae	1.4	2.6	2.0
Arctium spp.	0.1	0.0	0.1
Artemisia spp.	0.1	0.1	0.1
Aruncus dioicus	0.5	0.2	0.3
Asparagus officinalis	0.4	0.1	0.2
Asperula spp., *Cruciata* spp., *Galium* spp.*	0.0	0.0	0.0
Asteraceae	3.3	3.3	3.3
Atriplex spp., *Bassia* spp., *Chenopodium* spp., *Suaeda* spp.*	0.1	0.6	0.4
Atropa bella-donna	0.0	0.0	0.0
Begonia spp.	0.7	0.2	0.5
Berberidaceae	0.1	0.1	0.1

Betula spp.	0.3	0.1	0.2
Boraginaceae	0.0	0.0	0.0
Borago officinalis	0.0	0.0	0.0
Brassica spp.	2.3	2.2	2.2
Brassicaceae	2.4	3.4	2.9
Buddleja spp., *Cyclamen* spp., *Primula* spp.*	1.2	1.1	1.1
Buxus sempervirens	0.1	0.2	0.2
Calluna vulgaris	0.1	0.3	0.2
Calystegia spp.	0.0	0.0	0.0
Campanula spp., *Jasione* spp., *Legousia* spp., *Phyteuma* spp.*	0.1	0.0	0.0
Cannabis sativa	0.0	0.2	0.1
Carpinus betulus	0.1	0.0	0.0
Caryophyllaceae	0.1	0.1	0.1
Castanea sativa	1.4	1.2	1.3
Catalpa spp.	0.0	0.0	0.0
Centaurea jacea-form	0.8	0.3	0.6
Centaurea scabiosa	0.0	0.0	0.0
Cercis siliquastrum	0.0	0.0	0.0
Cirsium spp., *Carduus* spp., *Silybum* spp.*	0.5	0.7	0.6
Cistus spp.	0.0	0.0	0.0
Citrus spp.	0.0	0.0	0.0
Clinopodium vulgare	0.0	0.0	0.0
Colchicum autumnale	0.0	0.0	0.0
Colchicum spp.	0.0	0.0	0.0
Convolvulus spp.	0.1	0.1	0.1
Cornus sanguinea	0.2	0.5	0.3

Corylus avellana	0.0	0.0	0.0
Cotinus coggygria	0.2	0.1	0.1
Cotoneaster spp.	0.1	0.0	0.0
Crocus spp.	0.0	0.0	0.0
Cucurbita pepo	0.0	0.0	0.0
Cucurbitaceae	0.0	0.0	0.0
Cyanus montanus	0.0	0.0	0.0
Cyanus segetum	0.6	0.1	0.4
Cyanus triumfetti	0.0	0.1	0.0
Cyperaceae	0.1	0.1	0.1
Datura spp.	0.0	0.0	0.0
Datura stramonium	0.0	0.0	0.0
Datura suaveolens	0.0	0.0	0.0
Diervilla spp.	0.0	0.0	0.0
Echinops sphaerocephalus	0.0	0.0	0.0
Echium spp.	0.2	0.1	0.1
Elaeagnus angustifolia	0.0	0.0	0.0
Epilobium angustifolium	0.0	0.0	0.0
Epilobium spp.	0.0	0.0	0.0
Erica arborea	0.0	0.0	0.0
Erica carnea	0.0	0.0	0.0
Ericaceae	0.0	0.1	0.0
Euphorbia spp.	0.0	0.0	0.0
Fagopyrum esculentum	1.0	1.8	1.4
Fagus sylvatica	0.3	0.0	0.2
Filipendula spp.	1.0	0.7	0.9
Fragaria ananassa	0.1	0.1	0.1
Fragaria spp.	0.0	0.0	0.0
Fragaria vesca	0.1	0.0	0.1

Frangula alnus	0.0	0.0	0.0
Fraxinus excelsior	0.0	0.2	0.1
Fraxinus ornus	0.0	0.1	0.1
Genista spp.	0.0	0.0	0.0
Geranium spp.	0.1	0.1	0.1
Geum spp.	0.0	0.0	0.0
Gleditsia triacanthos	0.6	0.4	0.5
Hedera helix	4.3	2.5	3.4
Helianthemum spp.	0.1	0.2	0.2
Helianthus spp.	0.2	0.2	0.2
Helleborus niger	0.0	0.0	0.0
Helleborus spp.	0.0	0.0	0.0
Heracleum spp.	0.4	0.3	0.3
Hibiscus spp.	0.0	0.0	0.0
Hippocrepis spp.	0.0	0.1	0.1
Humulus lupulus	0.0	0.0	0.0
Hyacinthaceae	0.0	0.0	0.0
Hydrangea spp.	0.1	0.3	0.2
Hypericum spp.	0.6	1.2	0.9
Ilex aquifolium	0.0	0.0	0.0
Impatiens spp.	2.1	1.9	2.0
Iridaceae	0.0	0.0	0.0
Juglans spp.	0.1	0.1	0.1
Juncus spp., *Luzula* spp.*	0.2	0.2	0.2
Juniperus communis	0.0	0.0	0.0
Knautia spp.	0.1	0.0	0.1
Lamiaceae	0.0	0.0	0.0
Lamiaceae-form[2] *(6 Kst.)*	0.1	0.1	0.1
Lamiaceae-form[3] *(3 Kst.)*	0.6	0.1	0.3

Lantana camara	0.0	0.0	0.0
Lathyrus spp.	0.0	0.0	0.0
Leucojum vernum	0.0	0.0	0.0
Ligustrum vulgare	0.5	1.0	0.7
Lilium spp.	0.0	0.1	0.0
Linum spp.	0.0	0.0	0.0
Liriodendron tulipifera	0.1	0.1	0.1
Lonicera spp.	0.1	0.1	0.1
Loranthus europaeus	0.2	0.1	0.1
Lotus spp.	0.2	0.1	0.2
Lunaria rediviva	0.0	0.0	0.0
Lupinus spp.	0.0	0.0	0.0
Lysimachia spp.	0.0	0.2	0.1
Lythrum salicaria	0.0	0.3	0.2
Macleaya cordata	0.0	0.0	0.0
Malus spp., *Pyrus* spp., *Crataegus* spp.*	4.3	5.0	4.7
Malva-form[4]	0.0	0.0	0.0
Medicago spp.	0.0	0.1	0.1
Melampyrum spp.	0.7	0.6	0.7
Mercurialis spp.	0.0	0.0	0.0
Muscari spp.	0.0	0.0	0.0
Myosotis spp.	0.0	0.1	0.1
Narcissus spp.	0.0	0.0	0.0
not swollen	1.2	0.4	0.8
Nymphaea spp.	0.0	0.0	0.0
Ocimum basilicum	0.0	0.0	0.0
Oenothera tetragona	0.0	0.0	0.0
Onobrychis spp.	0.2	0.1	0.2

Ononis spinosa	0.0	0.0	0.0
Onopordum spp.	0.0	0.0	0.0
Ornithogalum spp.	0.0	0.0	0.0
Pachysandra terminalis	0.0	0.0	0.0
Paeonia spp.	0.0	0.0	0.0
Papaver rhoeas	0.6	0.3	0.4
Papaver somniferum	0.2	0.2	0.2
Papaver spp.	0.0	0.1	0.1
Parthenocissus spp.	1.6	2.6	2.1
Passiflora spp.	0.0	0.0	0.0
Persicaria bistorta	0.0	0.0	0.0
Persicaria maculosa	0.0	0.0	0.0
Petasites spp.	0.1	0.0	0.1
Petunia spp.	0.2	0.6	0.4
Phacelia tanacetifolia	0.9	1.8	1.4
Phlox spp.	0.0	0.0	0.0
Physocarpus monogynus	0.1	0.4	0.2
Picea abies, Abies alba	0.0	0.0	0.0
Pinus spp.	0.2	0.1	0.1
Plantago spp.	6.2	10.0	8.1
Platanus acerifolia	0.0	0.0	0.0
Poaceae	0.6	0.8	0.7
Polemonium caeruleum	0.0	0.0	0.0
Populus spp.	0.0	0.0	0.0
Potentilla spp.	0.7	0.2	0.4
Primula acaulis, P. elatior, P. veris	0.0	0.0	0.0
Prunus avium	0.7	0.0	0.4
Prunus domestica	0.0	0.1	0.1
Prunus padus	0.2	0.3	0.2

Prunus persica	0.0	0.0	0.0
Prunus spp.	1.8	2.1	1.9
Pterocarya fraxinifolia	0.0	0.0	0.0
Pulmonaria spp.	0.0	0.0	0.0
Quercus spp.	1.0	0.1	0.5
Ranunculus spp.	3.3	0.0	1.6
Rhamnus spp.	0.2	0.2	0.2
Rhododendron spp., *Vaccinium* spp.*	0.2	0.1	0.1
Ribes spp.	0.0	0.0	0.0
Robinia pseudacacia	0.1	0.3	0.2
Rosaceae	0.8	0.6	0.7
Rubiaceae	0.0	0.0	0.0
Rubus spp.	2.6	2.6	2.6
Rumex spp.	0.2	0.1	0.1
Salix spp.	5.4	6.0	5.7
Salvia glutinosa	2.0	1.6	1.8
Salvia officinalis	0.0	0.0	0.0
Sambucus nigra	0.0	0.0	0.0
Sambucus spp., *Philadelphus coronarius**	0.2	0.0	0.1
Sanguisorba officinalis	0.0	0.0	0.0
Scabiosa spp.	0.0	0.0	0.0
Scilla spp.	0.0	0.0	0.0
Sedum spp.	0.0	0.0	0.0
Sida hermaphrodita	0.0	0.0	0.0
Silphium perfoliatum	0.0	0.0	0.0
Sinapis spp.	3.4	3.2	3.3
Solanum spp.	0.0	0.0	0.0

Soldanella spp.	0.0	0.1	0.0
Sophora spp., *Linaria* spp.*	0.0	0.0	0.0
Symphytum spp.	0.0	0.0	0.0
Syringa vulgaris	0.3	0.3	0.3
Taraxacum-form[5]	4.7	3.1	3.9
Taxus baccata	0.0	0.0	0.0
Thuja spp.	0.0	0.0	0.0
Tilia spp.	0.5	0.1	0.3
Tragopogon spp.	0.0	0.0	0.0
Trifolium pratense-form[6]	3.1	2.1	2.6
Trifolium repens-form[7]	7.1	10.5	8.8
Ulmus spp.	0.0	0.0	0.0
unknown	1.9	1.4	1.7
unknown 223311	0.0	0.1	0.1
unknown 223321	0.0	0.0	0.0
unknown 223351	0.0	0.0	0.0
unknown 223361	0.4	0.2	0.3
unknown 333311	0.1	0.1	0.1
unknown 333361	0.1	0.5	0.3
unknown 334311	0.0	0.0	0.0
unknown 443111	0.1	0.0	0.0
unknown 443221	0.2	0.0	0.1
unknown 443251	0.0	0.1	0.1
unknown 443261	0.0	0.1	0.1
unknown 443311	0.0	0.1	0.1
unknown 443321	0.0	0.0	0.0
unknown 443361	0.1	0.0	0.1
unknown 553251	0.0	0.0	0.0
unknown 553261	0.1	0.0	0.1

unknown 553311	0.1	0.0	0.0
unknown 553321	0.0	0.0	0.0
unknown 553361	0.0	0.0	0.0
unknown 663261	0.1	0.0	0.0
unknown 663311	0.0	0.0	0.0
unknown 663361	0.0	0.0	0.0
unknown 773361	0.0	0.0	0.0
unknown 776131	0.0	0.0	0.0
Urtica spp., *Parietaria* spp.	0.0	0.0	0.0
Valeriana spp.	0.0	0.0	0.0
Verbascum spp.	0.0	0.1	0.0
Veronica spp.	0.0	0.0	0.0
Viburnum spp.	0.3	0.1	0.2
Vicia spp.	1.6	1.4	1.5
Vinca spp.	0.0	0.0	0.0
Viola tricolor	0.0	0.0	0.0
Viscum album	0.0	0.0	0.0
Vitis vinifera	0.0	0.2	0.1
Weigela spp.	0.0	0.0	0.0
Zea mays, *Triticum aestivum*	0.3	0.2	0.3

[1] *Achillea* spp., *Anthemis* spp., *Argyranthemum* spp., *Chrysanthemum* spp., *Leucanthemopsis* spp., *Leucanthemum* spp., *Tanacetum* spp., *Tripleurospermum* spp.

[2] *Acinos* spp., *Agastache* spp., *Clinopodium* spp., *Dracocephalum* spp., *Glechoma* spp., Lavandula spp., *Melissa* spp., *Mentha* spp., *Monarda* spp., *Nepeta* spp., *Ocimum* spp., *Origanum* spp., *Prunella* spp., *Rosmarinus* spp., *Salvia* spp., *Satureja* spp., *Thymus* spp.

[3] *Ajuga* spp., *Ballota* spp., *Betonica* spp., *Caryopteris* spp., *Galeobdolon* spp., *Galeopsis* spp., *Lamium* spp., *Leonorus* spp., *Marrubium* spp., *Melittis* spp., *Moluccella* spp., *Nepeta* spp., *Phlomis* spp., *Prasium* spp., *Scutellaria* spp., *Sideitis* spp., *Stachys* spp., *Teucrium* spp., *Vitex* spp.

[4] *Althaea* spp., *Dinacrusa hirsuta*, *Lavatera* spp., *Malva* spp.

[5] *Cicerbita* spp., *Cichorium* spp., *Crepis* spp., *Hieracium* spp., *Hypochaeris* spp., *Lactuca* spp., *Lapsana* spp., *Leontodon* spp., *Picris* spp., *Prenanthes* spp., *Reichardia* spp., *Scorzonera* spp., *Scorzoneroides* spp., *Sonchus* spp., *Taraxacum* spp., *Urospermum* spp., *Willemetia* spp.

[6] *T. pratense*, *T. hybridum*, *T. alexandrinum*, *T. incarnatum*

[7] *T. repens*, *T. montanum*, *T. nigrescens*

* Not further differentiable by light microscopy

Table S3: Number and frequency of partially (>50% comprised of one pollen type) and highly (>90%) monofloral pollen samples collected in Austria in 2014 and 2015 combined (n=1622 pollen samples). *Form cryptic groups, not further differentiable by light microscopy.

**>50%**			**>90%**		
**Pollen Type(s)**	**Amount of samples**	**Frequency [%]**	**Pollen Type(s)**	**Amount of samples**	**Frequency [%]**
Trifolium repens-form[1]	86	5.30	*Hedera helix*	18	1.11
Salix spp.	85	5.24	*Salix* spp.	10	0.62
Plantago spp.	72	4.44	*Trifolium repens*-form[1]	7	0.43
Hedera helix	53	3.27	*Impatiens* spp.	6	0.37
Malus spp., *Pyrus* spp., *Crataegus* spp.*	46	2.84	*Plantago* spp.	6	0.37
Sinapis spp.	41	2.53	*Sinapis* spp.	6	0.37
Acer spp.	35	2.16	*Vicia* spp.	5	0.31
Salvia glutinosa	29	1.79	*Castanea sativa*	3	0.18
Asteraceae	23	1.42	*Rubus* spp.	2	0.12
Brassica spp.	23	1.42	*Trifolium pratense*-form[2]	2	0.12
Impatiens spp.	21	1.3	*Acer* spp.	1	0.1
Parthenocissus spp.	21	1.3	*Hypericum* spp.	1	0.1
Phacelia tanacetifolia	21	1.3	*Salvia glutinosa*	1	0.1
Trifolium pratense-form[2]	19	1.2			
Brassicaceae	18	1.1			
Vicia spp.	18	1.1			
Castanea sativa	17	1.0			
Prunus spp.	15	0.9			
Taraxacum-form[3]	14	0.9			
Fagopyrum esculentum	13	0.8			
Hypericum spp.	12	0.7			
Rubus spp.	10	0.6			
Melampyrum spp.	9	0.6			
Apiaceae	8	0.5			
Buddleja spp., *Cyclamen* spp., *Primula* spp.*	7	0.4			
Ranunculus spp.	7	0.4			
Filipendula spp.	6	0.4			

Ligustrum vulgare	6	0.4
not swollen	6	0.4
Prunus avium	5	0.3
Asparagus officinalis	4	0.2
Begonia spp.	4	0.2
Aesculus hippocastanum	3	0.2
Atriplex spp., *Bassia* spp., *Chenopodium* spp., *Suaeda* spp.*	3	0.2
Gleditsia triacanthos	3	0.2
Lythrum salicaria	3	0.2
Papaver rhoeas	3	0.2
Poaceae	3	0.2
unknown 333361	3	0.2
Achillea-form[4]	2	0.1
Anemone spp., *Clematis* spp., *Pulsatilla* spp., *Ranunculus* spp.*	2	0.1
Calluna vulgaris	2	0.1
Cannabis sativa	2	0.1
Papaver somniferum	2	0.1
Physocarpus monogynus	2	0.1
Potentilla spp.	2	0.1
Robinia pseudacacia	2	0.1
Amorpha fruticosa	1	0.1
Betula spp.	1	0.1
Cotinus coggygria	1	0.1
Cyanus segetum	1	0.1
Heracleum spp.	1	0.1
Hydrangea spp.	1	0.1
Loranthus europaeus	1	0.1
Medicago spp.	1	0.1
Papaver spp.	1	0.1
Petunia spp.	1	0.1
Pinus spp.	1	0.1

Prunus padus	1	0.1
Sambucus spp., *Philadelphus coronarius**	1	0.1
Tilia spp.	1	0.1
unknown 223361	1	0.1
unknown 333311	1	0.1
unknown 443221	1	0.1
unknown 443261	1	0.1
Vitis vinifera	1	0.1
Zea mays, *Triticum aestivum*	1	0.1

[1] *T. repens*, *T. montanum*, *T. nigrescens*

[2] *T. pratense*, *T. hybridum*, *T. alexandrinum*, *T. incarnatum*

[3] *Cicerbita* spp., *Cichorium* spp., *Crepis* spp., *Hieracium* spp., *Hypochaeris* spp., *Lactuca* spp., *Lapsana* spp., *Leontodon* spp., *Picris* spp., *Prenanthes* spp., *Reichardia* spp., *Scorzonera* spp., *Scorzoneroides* spp., *Sonchus* spp., *Taraxacum* spp., *Urospermum* spp., *Willemetia* spp.

[4] *Achillea* spp., *Anthemis* spp., *Argyranthemum* spp., *Chrysanthemum* spp., *Leucanthemopsis* spp., *Leucanthemum* spp., *Tanacetum* spp., *Tripleurospermum* spp.

* Not further differentiable by light microscopy
